# Vaccination coverage, hesitancy and associated factors: a household survey of a cohort of children born in 2017 and 2018 in urban areas of state capital cities in the Brazilian Northeast

**DOI:** 10.1590/S2237-96222024v33e20231298.especial2.en

**Published:** 2024-11-01

**Authors:** Ramon da Costa Saavedra, Martha Suely Itaparica de Carvalho Santiago, Maria da Glória Lima Cruz Teixeira, Maria Bernadete de Cerqueira Antunes, Rejane Christine de Sousa Queiroz, Luisa Helena de Oliveira Lima, Alberto Novaes Ramos, Anderson Fuentes Ferreira, Adjoane Mauricio Silva Maciel, Jaqueline Caracas Barbosa, Ana Paula França, Carla Magda Allan Santos Domingues, José Cássio de Moraes, ICV 2020 Group, Adriana Ilha da Silva, Alberto Novaes Ramos, Ana Paula França, Andrea de Nazaré Marvão Oliveira, Antonio Fernando Boing, Carla Magda Allan Santos Domingues, Consuelo Silva de Oliveira, Ethel Leonor Noia Maciel, Ione Aquemi Guibu, Isabelle Ribeiro Barbosa Mirabal, Jaqueline Caracas Barbosa, Jaqueline Costa Lima, José Cássio de Moraes, Karin Regina Luhm, Karlla Antonieta Amorim Caetano, Luisa Helena de Oliveira Lima, Maria Bernadete de Cerqueira Antunes, Maria da Gloria Teixeira, Maria Denise de Castro Teixeira, Maria Fernanda de Sousa Oliveira Borges, Rejane Christine de Sousa Queiroz, Ricardo Queiroz Gurgel, Rita Barradas Barata, Roberta Nogueira Calandrini de Azevedo, Sandra Maria do Valle Leone de Oliveira, Sheila Araújo Teles, Silvana Granado Nogueira da Gama, Sotero Serrate Mengue, Taynãna César Simões, Valdir Nascimento, Wildo Navegantes de Araújo

**Affiliations:** 1 Universidade Federal da Bahia, Instituto de Saúde Coletiva, Salvador, BA, Brazil; 2 Universidade de Pernambuco, Faculdade de Ciências Médicas, Recife, PE, Brazil; 3 Universidade Federal do Maranhão, Departamento de Saúde Pública, São Luís, MA, Brazil; 4 Universidade Federal do Piauí, Campus Senador Helvídio Nunes de Barros, Picos, PI, Brazil; 5 Universidade Federal do Ceará, Faculdade de Medicina, Departamento de Saúde Comunitária, Fortaleza, CE, Brazil; 6 Universidade Federal do Ceará, Faculdade de Medicina, Programa de Pós-graduação em Saúde Pública, Fortaleza, CE, Brazil; 7 Faculdade de Ciências Médicas da Santa Casa de São Paulo, São Paulo, SP, Brazil; 8 Pan American Health Organization, Brasília, DF, Brazil; Universidade Federal do Espírito Santo, Vitória, ES, Brazil; Universidade Federal do Ceará, Departamento de Saúde Comunitária, Fortaleza, CE, Brazil; Faculdade Ciências Médicas Santa Casa de São Paulo, São Paulo, SP, Brazil; Secretaria de Estado da Saúde do Amapá, Macapá, AP, Brazil; Universidade Federal de Santa Catarina, SC, Brazil; Organização Pan-Americana da Saúde, Brasília, DF, Brazil; Instituto Evandro Chagas, Belém, PA, Brazil; Faculdade de Ciências Médicas Santa Casa de São Paulo, Departamento de Saúde Coletiva, São Paulo, SP, Brazil; Universidade Federal do Rio Grande do Norte, Natal, RN, Brazil; Universidade Federal do Ceará, Departamento de Saúde Comunitária, Fortaleza, CE, Brazil; Universidade Federal de Mato Grosso, Cuiabá, MT, Brazil; Universidade Federal do Paraná, Curitiba, PR, Brazil; Universidade Federal de Goiás, Goiânia, GO, Brazil; Universidade Federal do Piauí, Teresina, PI, Brazil; Universidade de Pernambuco, Faculdade de Ciências Médicas, Pernambuco, PE, Brazil; Instituto de Saúde Coletiva, Universidade Federal da Bahia, Salvador, BA, Brazil; Secretaria de Estado da Saúde de Alagoas, Maceió, AL, Brazil; Universidade Federal do Acre, Rio Branco, AC, Brazil; Universidade Federal do Maranhão, Departamento de Saúde Pública, São Luís, MA, Brazil; Universidade Federal de Sergipe, Aracaju, SE, Brazil; Secretaria Municipal de Saúde, Boa Vista, RR, Brazil; Fundação Oswaldo Cruz, Mato Grosso do Sul, Campo Grande, MS, Brazil; Fundação Oswaldo Cruz, Escola Nacional de Saúde Pública Sergio Arouca, Rio de Janeiro, RJ, Brazil; Universidade Federal do Rio Grande do Sul, Porto Alegre, RS, Brazil; Fundação Oswaldo Cruz, Instituto de Pesquisa René Rachou, Belo Horizonte, MG, Brazil; Secretaria de Desenvolvimento Ambiental de Rondônia, Porto Velho, RO, Brazil; Universidade de Brasília, Brasília, DF, Brazil

**Keywords:** Cobertura de Vacunación, Vacilación a la Vacunación, Vacunación Masiva, Programas de Inmunización, Encuesta epidemiológica, Vaccination Coverage, Vaccine Hesitancy, Mass Vaccination, Immunization Programs, Health Surveys

## Abstract

**Objective:**

To estimate vaccination coverage and analyze sociodemographic factors associated with non-vaccination in children born in 2017 and 2018 in the state capitals of Northeast Brazil.

**Methods:**

A household survey using cluster sampling was conducted from 2020-2022 to estimate vaccination coverage and hesitancy. Factors associated with non-vaccination were analyzed using logistic regression to calculate Odds Ratios (OR) and their Confidence Intervals (95%CI).

**Results:**

Natal was the capital with the lowest vaccination coverage, below 75.0% for most immunizers. Teresina had rates equal to or greater than 90.0% for all vaccines. Among those interviewed, 99.1% (95%CI 98.9;99.3) believe that vaccines are important for health; 95.4% (95%CI 95.0;95.8) trust immunobiologicals and 79.6% (95%CI% 78.8;80.3) are not afraid of reactions. Belonging to the highest socioeconomic stratum (adjusted OR: 1.34 – 95%CI 1.20;1.50) was as a factor associated with non-vaccination.

**Conclusion:**

Low coverage highlights the need for a better understanding of regional specificities and social inequalities.

## Study contributions

**Main results:**

Three northeastern capitals achieved targets for just two or three vaccines. The majority of interviewees expressed favorable opinions regarding the National Immunization Program. However, a third of those interviewed reported that they had sought vaccination but had been unable to vaccinate their children.

**Implications for services:**

Rethinking immunization strategies in the Brazilian National Health System, to adapt them to regional contexts. Operational obstacles related to the supply of vaccines require special attention, in addition to integrated health communication and education actions.

**Perspectives:**

Understanding the regional specificities presented will support the National Immunization Program in implementing strategies contextualized to territories, via microplanning of vaccination actions, to recover vaccination coverage.

## RESUMEN

**Objetivo:**

Estimar cobertura vacunal, analizar factores sociodemográficos asociados a la no vacunación en niños nacidos vivos en 2017/2018 en capitales del noreste de Brasil.

**Métodos:**

Se realizó encuesta de hogares con muestreo por conglomerados, entre 2020/ 2022, para estimar cobertura y reticencias a vacunarse. Factores asociados a bajas coberturas de vacunación se analizaron mediante regresión logística calculando los odds ratios (OR) y sus intervalos de confianza (IC del 95%).

**Resultados:**

La capital con menor cobertura de vacunación: Natal, con < 75,0% para la mayoría de inmunizadores; Teresina presentó cobertura ≥ 90,0% en todos inmunizadores. 99,1% (IC_95_% 98,9%;99,3%) cree que vacunas son importantes para la salud; 95,4% (IC_95_% 95,0%;95,8%) confía en los inmunobiológicos distribuidos por gobierno y 79,6% (IC_95_% 78,8%;80,3%) no teme reacciones. Ser residente en zona de mayor estrato socioeconómico (Orajustado:1,34 - IC_95_% 1,20;1,50) se identificó como factor asociado a no vacunación.

**Conclusión:**

Bajas coberturas destacan la necesidad de comprender mejor las especificidades regionales y desigualdades sociales.

## INTRODUCTION

Investment in sustainable actions to expand vaccination coverage has contributed to the control of vaccine-preventable diseases in Brazil and around the world, reducing morbidity and mortality from these infections and increasing life expectancy.^1,2^ In the Northeast region of Brazil, the expansion of the Family Health Strategy and income transfer initiatives have also been important for improving vaccination coverage.^3,4^

Intensification of immunization actions in the Brazilian Northeast has had a positive impact on the population’s health conditions. After the establishment of the National Plan to Eliminate Measles (1992), the state of Ceará spent 13 years (2000-2013) without recording any cases of the disease, between 2000 and 2013.^5^ The introduction of the oral vaccine against human rotavirus (2006) reduced hospitalizations and deaths of children in the Northeast,^6,7^ with the greatest drops seen in Recife-PE (77% between 2006 and 2007).^8^ The introduction of the meningococcal C vaccine (2010) led to a notable decrease in cases of meningococcal meningitis due to this serogroup in the region – approximately 80%, between 2010 and 2018.^9^

Despite the advances achieved in decades of coordinated work between the three public spheres of management of the Brazilian National Health System (*Sistema* Único *de Saúde* - SUS), as of 2016, there has been a significant decline in vaccination coverage of the main immunization agents recommended for children. In the Northeast, this reduction has been even greater in children under 1 year old, compared to other Brazilian regions.^9-11^

Several hypotheses have been raised to explain this critical scenario, which should not be attributed to a single factor. Elimination of some vaccine-preventable diseases has changed the risk perception of a considerable portion of the population. A false sense of security has contributed to the failure to recognize vaccination as a necessary intervention for protecting health.^12,13^ Misinformation, associated with the dissemination of fake news by anti-vaccine movements, is another relevant factor.^14^ Recent research showed that Brazil is experiencing an “epidemic of misinformation” about vaccines.^15^

In this context, vaccination hesitancy emerges as a worrying phenomenon. In addition to simply refusing vaccination, operational and structural aspects of immunization services need to be better understood.^16,17^ In 2019, the World Health Organization (WHO) listed ten global health threats and included vaccination hesitancy among the priorities to be addressed.^18^ Understanding these phenomena and identifying how they influence vaccination coverage is essential for providing evidence that supports the design of effective public policies that are contextualized to regional specificities.

Vaccination coverage rates reflect the population’s adherence to the immunization program, the existence of people at risk of vaccine-preventable diseases and the effectiveness of health services.^19^ Considering that carrying out surveys to assess vaccination coverage is a relevant action, this study aims to estimate vaccination coverage and analyze sociodemographic factors associated with non-vaccination in children born 2017 and 2018 in the state capital cities of Northeast Brazil.

## METHODS

### Study design

This is a household survey conducted between 2020 and 2022, using cluster sampling based on a cohort of children born alive in 2017 and 2018, in urban areas of the state capital cities of Northeast Brazil. This is a selected part taken from the *Vaccination Coverage Survey in the capital cities of 26 states, the Federal District and 12 interior region municipalities of children born in 2017-2018 living in urban areas*, details of the methods and operational aspects of which have been presented previously in an article.^20^

### Background

The Northeast region of Brazil covers an area of 1,558,000 km² and has an estimated population of 54,644,582 inhabitants (26.9% of the Brazilian population). The Northeast region capital cities have 11,385,286 inhabitants, whereby Aracaju (602,757) has the smallest population and Fortaleza (2,428,708) has the largest.^21^

### Population and data source 

The study population is made up of children born in 2017 and 2018, residing in the nine state capital cities of the Northeast region, according to the Live Birth Information System (*Sistema de Informação de Nascidos Vivos* - SINASC). The data source was the national survey mentioned above.

### Sampling procedure

The study included children whose vaccination trajectories were analyzed from birth to 24 months of age, whose addresses, as per the SINASC, were georeferenced in census tracts of residence and grouped into clusters formed by four ecological strata (A, B, C , D) defined by socioeconomic characteristics, in which A presents the best living conditions, and D, the poorest.

The census tracts of each city were used to define the strata, according to the 2010 Demographic Census, classified based on the average income of the heads of household, the proportion of literate heads of household and the proportion of heads of household with income greater than or equal to 20 minimum wages.

Random selection of the expected number of children in each stratum was performed taking the clusters. Sample size in the strata varied according to the number of surveys carried out in the municipalities, and was defined according to guidelines established in the published article which describes the methodology.^20^

### Data collection and variables

Household visits took place from 2020 to 2022, according to the addresses identified on the SINASC. A questionnaire was used to collect information on the following sociodemographic characteristics:

- Family: household crowding (yes/no), grandparent living in the household (yes/no), access to social benefits (yes/no) and monthly income (up to BRL 1,000, BRL 1,001-BRL 3,000, BRL 3,001-BRL 8,000, and more than BRL 8,000);
- Mother of the child: age group (< 20 years, 20-34 years, 35 years or over), race/skin color (White, Black, mixed race, Asian, Indigenous), schooling (years of study: 0-8, 9-12, 13-15, 16 or more), having a job (yes/no), living with a partner (yes/no), number of children alive;
- Child: sex (male/female), race/skin color, attends daycare (yes/no), type of child delivery (normal/cesarean), birth order.

Regarding immunization, information was collected regarding type of vaccine and number of doses received. The full vaccination schedule considered in this study, up to 24 months of life, was that recommended by the National Immunization Program (*Programa Nacional de Imunizações* - PNI), which includes administration of the following vaccines: Bacillus Calmette-Guérin (BCG) and hepatitis B, at birth; 5-in-1 (against diphtheria, tetanus, pertussis, hepatitis B and *Haemophilus influenzae B*) and inactivated poliovirus vaccine (IPV), at 2, 4 and 6 months; pneumococcal 10-valent and human rotavirus vaccine, at 2 and 4 months; yellow fever vaccine, at 9 months; meningococcal C conjugate vaccine, at 3, 5 and 12 months; MMR vaccine (against measles, mumps and rubella), at 12 and 15 months; and adsorbed diphtheria, tetanus and pertussis (DTP) vaccine, hepatitis A, oral poliovirus vaccine (OPV) and varicella, at 15 months old.

Vaccination hesitancy was analyzed from the viewpoint of those responsible for the children, addressing questions about beliefs (confidence in vaccines), fear of reactions, importance of vaccination and the decision to vaccinate (or not). The answers were interpreted as unfavorable, indifferent or favorable to the actions proposed by the PNI. Personal issues (difficulties in taking the child to be vaccinated) and operational issues (barriers to accessing services) that could make vaccination difficult were also raised.

Finally, vaccination status was verified by means of the photographic record of the child’s vaccination card.

### Statistical methods

Vaccination coverage indicators were calculated considering the total number of last doses of the schedule for each immunizing agent received (numerator) and the sample population (denominator), multiplied by 100. The targets of the National Child Vaccination Calendar were used as a reference, namely: 90% for BCG and human rotavirus vaccines; 95% for other vaccines. In this study, the analyses did not include yellow fever vaccine, because in 2017 and 2018 there was no recommendation for this immunobiological product for most states in the Northeast. 

Weighted estimates of vaccination coverage and confidence intervals (95%CI) were calculated for each vaccine, using statistical significance of p < 0.05.^21^

Exploratory analysis was performed to identify risk factors associated with non-vaccination, using logistic regression, with calculation of the crude odds ratio (ORc) and 95% confidence intervals (95%CI). Variables that showed association with p < 0.20 in the univariate regression were included in the model, with calculation of the adjusted odds ratio (OR-a), using the stepwise method. Model collinearity was checked for by analysis of the variance inflation factor, excluding those variables with presence of this factor from the model. For the dependent variable – vaccination status at 24 months of age – dichotomization was carried out as *fully vaccinated* (reference), considering children who had received all vaccination schedule doses, or *not fully vaccinated*, for those lacking one or more valid doses of the vaccination schedule.

The analyses were conducted using R® software version 4.2.2, using the tidyverse package; Stata® version 13 and Microsoft Office Excel®.

### Ethical considerations

The study was approved by the Research Ethics Committee of the *Instituto de Saúde Coletiva da Universidade Federal da Bahia*, as per Opinion No. 3.366.818, on June 4, 2019, and Certificate of Submission for Ethical Appraisal (*Certificado de Apresentação de Apreciação* Ética - CAAE) No. 4306919.5.0000.5030; and by the Research Ethics Committee of the *Irmandade da Santa Casa de São Paulo*, as per Opinion No. 4.380.019, on November 4, 2020, and CAAE No. 39412020.0.0000.5479.

## RESULTS

A total of 10,290 interviews were carried out, distributed proportionally between socioeconomic strata and cities. Of this total, 2,249 (21.9%) belonged to stratum A; 2,659 (25.8%), to stratum B; 2,677 (26.0%), to stratum C; and 2,705 (26.3%), to stratum D. The largest number of interviews occurred in Salvador (1,818; 17.7%), Recife (1,689; 16.4%) and Fortaleza (1,612; 15.7%); while the lowest number of interviews occurred in Natal (685; 6.7%). The children’s vaccination card was provided by 99.2% of families for photographing. Use of private vaccination services at least once was identified in 15.0% of the interviews (Table 1).

**Table 1:** Proportion (%) of the sociodemographic characteristics of the families of children born in 2017-2018, by state capital cities of the Northeast region of Brazil, Vaccination Coverage Survey 2020-2022 (n = 10,290)

**Characteristics**	**Aracaju**	**Fortaleza**	**João Pessoa**	**Maceió**	**Natal**	**Recife**	**Salvador**	**São Luís**	**Teresina**	**Total**
**Family characteristics**										
**Household crowding**	5.0	9.8	4.3	8.6	8.0	9.9	12.2	8.4	13.6	9.3
*Bolsa Família* **beneficiary**	23.1	51.7	28.9	40.4	31.2	32.6	36.6	36.3	32.0	36.0
**Grandparent present in household**	47.8	41.1	31.1	37.1	55.0	42.3	36.8	63.7	73.6	30.8
**Monthly family income**										
Up to BRL 1,000	18.0	38.8	21.0	46.8	36.9	37.8	45.2	29.6	14.7	34.1
BRL 1,001 - BRL 3,000	31.1	43.3	46.9	23.5	32.6	24.9	30.9	37.9	43.8	34.4
BRL 3,001 - BRL 8,000	24.8	9.4	20.1	12.8	16.1	13.0	8.0	13.3	18.2	13.9
BRL 8,001 or more	13.9	3.8	11.1	5.3	6.3	9.4	9.4	7.8	13.0	8.7
Unable to answer/did not answer	12.2	4.7	0.9	11.6	8.2	14.9	6.4	11.2	10.2	8.9
**Maternal characteristics**										
**Schooling (years of study)**										
0-8	9.4	10.1	8.1	19.5	13.7	12.1	13.3	5.4	10.3	11.5
9-12	12.7	25.5	12.9	18.6	13.9	16.5	20	11.4	13.7	17.2
13-15	39.6	47.5	41.4	40.2	38.7	45.9	41.4	50.4	41.2	43.4
16 or more	33.7	14.3	36.4	17.4	31.8	23.7	22.7	27.2	33	25.1
Unable to answer/did not answer	4.7	2.7	1.2	4.3	1.9	1.8	2.6	5.7	1.8	2.8
**Age when child born (in years)**										
< 20	3.1	2.5	3.4	5.7	2.2	3.3	2.4	3	3.4	3.1
20-34	58.4	64.5	56.3	70.4	59.6	62.5	56.5	67	61.3	61.6
35 or over	38.2	30.9	40.2	23.5	38.2	33.9	40.9	29.6	34.8	34.7
Unable to answer/did not answer	0.2	2.1	0.1	0.4	0	0.2	0.2	0.4	0.4	50
**Race/skin color**										
White	23.6	20.2	34.2	36.9	40.9	30.8	13.4	23.7	21.6	25.5
Black	12.8	5.5	4.3	8.5	12.3	12.8	40	16.5	16	15.9
Mixed race	57.6	71.5	59.2	52.4	43.5	54.2	42.3	54.2	60.5	55.2
Asian	1.2	0.4	0.9	0.2	1.5	0.4	1.7	0.5	0	0.8
Indigenous	0.2	0.1	0.2	0.2	0.1	0.2	0.2	0.1	0	20
Unable to answer/did not answer	4.7	2.3	1.2	1.7	1.8	1.6	2.5	5	1.9	2.4
**Has a paid job**	56.1	52	46.8	41	47.7	41.8	46.9	43.8	50.2	47.2
**Lives with a partner**	70.6	70.5	80.6	66.2	70.5	68	65.1	67.9	70.5	69.4
**Number of children (average)**	1.89	2.01	2.09	2.02	1.98	2.07	2.06	1.96	1.98	2.02
**Children’s characteristics**										
**Sex**										
Male	51.6	53.1	51.2	50.2	53.9	49.6	51.6	52.8	48.3	51.3
Female	48.4	46.9	48.8	49.8	46.1	50.4	48.4	47.2	51.7	48.7
**Birth order**										
First	52.8	48.3	50.6	48.2	49.6	47	49.4	50.5	49.6	49.3
Second	29.6	31.9	29.1	33.6	29.6	31.1	28.7	31.6	32.3	30.8
Third	10.9	13.3	12.8	12.5	13.1	13.1	12.2	11.4	11.8	12.4
Fourth or more	6.3	6.5	7.5	5.5	7.6	8.8	9.7	6.6	6.3	7.5
**Race/skin color**										
White	33	30.4	40.5	39.8	55.6	41.3	19.3	33.3	30.9	34.1
Black	7.8	2.9	1.5	4.1	4.4	6	30.1	8.8	10.8	9.9
Mixed race	57.8	66.4	57.3	55.9	39.3	52.2	48.6	57.6	58.2	55.2
Asian	1	0.2	0.3	0.2	0.6	0.4	1.8	0.2	0	60
Indigenous	0.2	0.1	0.1	0	0	0	0	0.1	0	0
Unable to answer/did not answer	0.2	0	0.2	0	0.1	0.1	0.3	0	0.1	10
**Attends daycare/school**	45	44.4	29.2	27.6	46.3	22.8	31.3	27.5	27.5	33
**Type of child delivery**										
Normal (vaginal)	43.8	40.6	34.5	39.5	35.6	49.4	52.9	43.2	33.6	43.1
Cesarean	56	59.2	65.4	59.5	64.4	50.5	47	56.6	66.4	56.7
Unable to answer	0.2	0.2	0.1	1	0	0.1	0.1	0.2	0	0.2
**Has a vaccination card**	99.6	99.3	99.6	97.4	99.4	98.8	99.3	99.2	100	99.2
**Used a private service**	23.7	11.5	15.9	7.4	19.1	15.7	14.2	16.7	14.9	15

We found that there was household crowding in 9.3% of households (more than three people per bedroom), and 36.0% of families had access to social benefits. Monthly family income of up to BRL 1,000.00 was found for 34.1% of interviewees, with a higher proportion in stratum D (53.4%) and a lower proportion in stratum A (12.9%). Maceió had the largest share of families in this income range (46.8%). Only 5.7% reported monthly income greater than BRL 8,000.00, being concentrated in stratum A (29.7%) and in Aracaju (13.9%) (Table 1).

Mothers of mixed race or Black race/skin color (71.1%) predominated, ranging from 55.8% (Natal) to 82.3% (Salvador). Mothers of White race/skin color were most frequently reported in stratum A (40.0%), while those of Black race/skin color were most frequently reported in stratum D (20.1%). The majority of mothers (43.4%) had completed between 13 and 15 years of formal education, mainly in strata C (52.0%) and D (47.7%) (Table 1).

The majority of children were male (52.3%), mainly in strata B and C. In stratum A (50.5%) and in the cities of Teresina (51.7%) and Recife (50.4%), the majority were female. Mixed race children (55.2%) predominated overall, and in Fortaleza accounted for 66.4%. The highest proportion of children of White race/skin color was found in Natal (55.6%), above the overall percentage (34.1%) (Table 1).

In the analysis of vaccination coverage, four state capitals achieved the target for at least one product: Recife [BCG, 93.2% (95%CI 91.7;94.7)]; Salvador [BCG, 95.3% (95%CI 94.4;96.38); pneumococcal 10, 95.4% (95%CI 94.4;96.3)], São Luís [BCG, 95.1% (95%CI 93.6;96.6); pneumococcal 10, 95.2% (95%CI 93.7;96.6)] and Teresina [BCG, 94.9% (95%CI 93.4;96.3); pneumococcal 10, 97.3% (95%CI 96.3;98.4); human rotavirus, 90,3% (95%CI 88.4;92.3); meningococcal C, 96.0% (95%CI 94.7;97.2); MMR, 97.1% (95%CI 96.0;98.2); hepatite A, 95.6% (95%IC 94.2;97.0)]. The remaining state capitals did not achieve the targets for any of the vaccines. Natal had lower vaccination coverage, with an overall average of 77.3%; on the other hand, Teresina had the best indicators, both in the joint average (93.9%) and in the analysis by vaccine, being the only city where all vaccines had coverage greater than or equal to 90% (Table 2).

**Table 2:** Vaccination coverage (%) and 95% confidence intervals for vaccines among children born in 2017-2018 in state capital cities of the Northeast region of Brazil, Vaccination Coverage Survey 2020-2022 (n = 10,290)

	**Brazil** ^a^	**Aracaju**	**Fortaleza**	**João Pessoa**	**Maceió**	**Natal**	**Recife**	**São Luís**	**Salvador**	**Teresina**
**Single dose of Bacillus Calmette-Guérin**	89.6 (89.0;90.2)	85.3 (82.8;87.8)	87.5 (85.6;89.4)	89.5 (87.1;91.9)	86.5 (84.0;89.1)	70.9 (67.7;74.2)	93.2 (91.7;94.7)	95.1 (93.6;96.6)	95.3 (94.4;96.3)	94.9 (93.4;96.3)
**Single dose of hepatitis B**	88.7 (88.1;89.3)	85.5 (83.1;87.9)	86.1 (84.3;87.9)	88.7 (86.4;91.0)	83.2 (80.7;85.7)	70.8 (67.6;74.2)	92.7 (91.3;94.0)	94.2 (92.6;95.7)	93.7 (92.6;94.8)	94.3 (92.8;95.8)
**Second dose of pneumococcal**	90.3 (89.7;90.9)	87.4 (85.2;89.6)	87.9 (86.0;89.8)	88.8 (86.5;91.1)	86.2 (83.7;88.7)	86.6 (83.5;89.7)	91.8 (90.5;93.1)	95.2 (93.7;96.6)	95.4 (94.4;96.3)	97.3 (96.3;98.4)
**Second dose of human rotavirus**	82.0 (81.3;82.7)	84.3 (81.9;86.7)	77.7 (75.7;79.9)	76.1 (73.5;78.8)	76.4 (73.8;79.0)	68.3 (64.9;71.7)	86.0 (84.3;87.7)	82.4 (79.9;85.0)	85.1 (83.5;86.7)	90.3 (88.4;92.3)
**Second dose of meningococcal C**	89.3 (88.7;89.9)	87.8 (85.6;90.0)	85.6 (83.7;87.4)	87.0(84.5;89.5)	85.8 (83.4;88.2)	84.3 (81.1;87.4)	89.9 (88.5;91.3)	94.0 (92.4;95.6)	94.9 (93.8;95.9)	96.0 (94.7;97.2)
**Third dose of 5-in-1**	87.9 (87.3;88.5)	86.9 (84.7;89.1)	83.5 (81.6;85.4)	83.7 (81.1;86.3)	83.9 (81.5;86.3)	76.2 (73.0;79.4)	90.1 (88.7;91.5)	91.8 (89.9;93.6)	94.0 (92.9;95.1)	92.5 (90.8;94.3)
**Third dose of poliovirus**	87.8 (87.2;88.4)	87.1 (84.8;89.4)	83.7 (81.8;85.6)	86.1 (83.6;88.6)	85.3 (82.8;87.8)	73.7 (70.6;76.8)	85.1 (83.3;86.7)	94.0 (92.4;95.6)	94.0 (92.9;95.1)	92.9 (91.2;94.6)
**First dose of MMR**	90.8 (90.2;91.4)	88.4 (86.1;90.7)	88.1 (86.3;90.0)	89.5 (87.3;91.8)	84.1 (81.6;86.6)	84.1 (80.9;87.3)	92.3 (91.0;93.6)	92.5 (90.7;94.3)	94.6 (93.6;95.6)	97.1 (96.0;98.2)
**Single dose of hepatitis A**	88.1 (87.5;88.7)	86.3 (84.0;88.7)	86.0(84.1-87.9)	83.8 (81.4-86.2)	82.9 (80.4-85.4)	81.4 (78.2-84.6)	88.4 (86.9-89.9)	86.5 (84.2-88.8)	92.4 (91.2-93.6)	95.6 (94.2-97.0)
**Diphtheria, tetanus and pertussis booster**	83.9 (83.2;84.6)	83.6 (81.2;85.9)	78.8 (76.6;81.0)	75.1 (72.5;77.7)	76.6 (74.0;79.2)	74.3 (71.2;77.5)	84.3 (82.6;86.0)	84.7 (82.2;87.1)	91.7 (90.4;93.0)	89.9 (87.9;91.8)
**Single dose of varicella**	86.9 (86.2;87.6	86.3 (84.1;88.4)	85,0 (83.1;86.9)	79.9 (77.4;82.4)	82.9 (80.4;85.4)	79.4 (76.2;82.6)	88.5 (87.0;90.0)	82.7 (80.1;85.2)	91.4 (90.1;92.7)	92.5 (90.8;94.3)

a) Average of the Brazilian state capitals and Federal District. Target achieved (90% for BCG and rotavirus; 95% for the remainder) 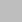
Target not achieved (90%-94.9%, except BCG and rotavirus) 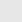
Target not achieved (85%-89.9%) 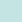
Target not achieved (80%-84.9%) 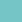
Target not achieved (75%-79.9%) 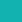
Target not achieved (70%-74.9%) 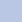
Target not achieved (below 70%) 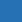

**Figure 1:**
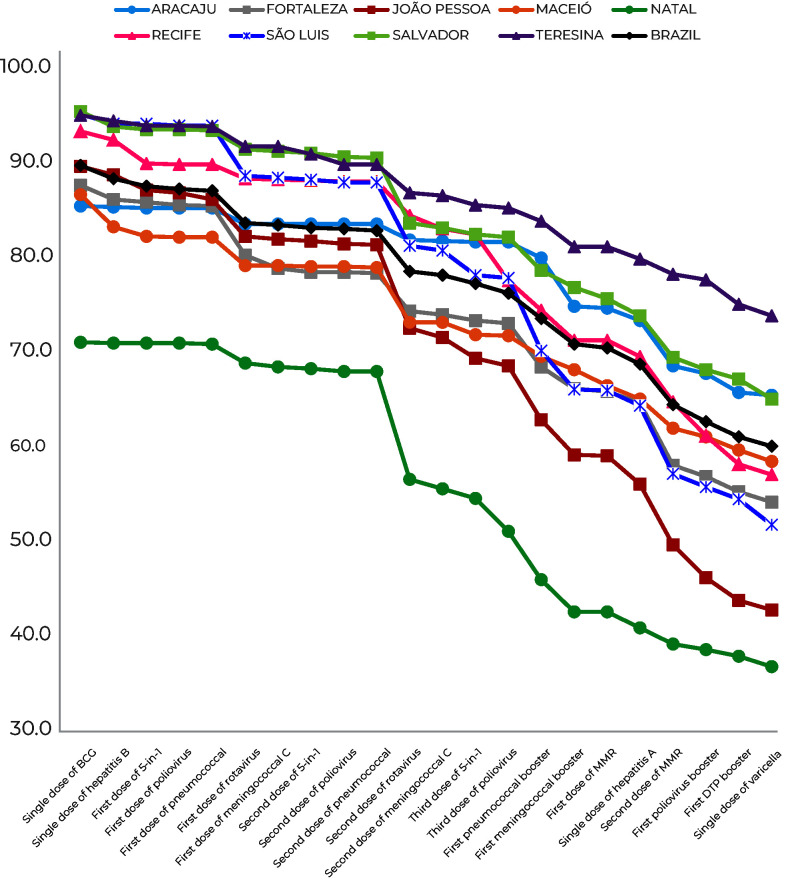
Evolution of vaccination coverage among children born in 2017-2018, by vaccines and state capital cities of the Northeast region of Brazil, Vaccination Coverage Survey 2020-2022 (n = 10,290)

With regard to the evolution of vaccination coverage, depending on the sequence provided for by the vaccination calendar, heterogeneous behavior was found between the state capitals, with three of them (Teresina, Salvador and Aracaju) performing better than the national average; a further three (Fortaleza, João Pessoa and Natal) performed below the national average; and the remaining cities (Recife, São Luís and Aracaju) were in the same range as the national vaccination coverage. Natal stood out as the capital city with the poorest performance, the highest being 70.9% for BCG and the lowest being 36.6% for varicella (Figure 1).

In the perception of parents/guardians regarding issues involving vaccination hesitancy, 99.1% (95%CI 98.9;99.3) believed that vaccines are important for children’s health, with little variation between strata. Regarding the need to be vaccinated against diseases that may no longer exist, 80.7% (95%CI 79.9;81.4) considered it necessary to maintain vaccination. 98.6% (95%CI 98.3;98.7) of parents/guardians agreed with the statement that “vaccinating children is important for the health of children in the neighborhood”, with no significant variation between strata and capital cities.

**Figure 2:**
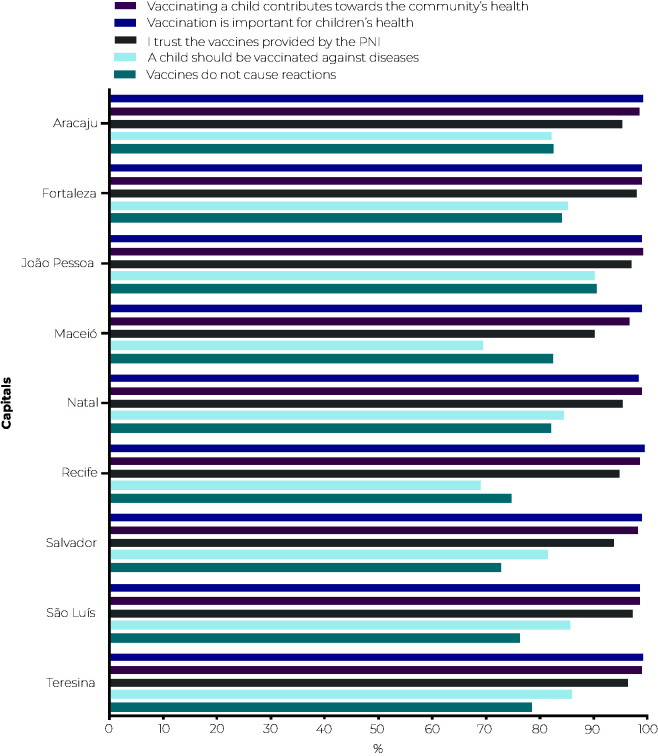
Perception regarding vaccines, according to parents and guardians of children born in 2017 and 2018, in the state capital cities of the Northeast region of Brazil, Vaccination Coverage Survey 2020-2022 (n = 10,290)

Regarding the possibility of adverse reactions occurring after vaccines being administered, 20.4% (95%CI 19.6;21.2) of those interviewed said they believe that reactions occur due to vaccines. In stratum D, 20.2% (95%CI 18.8;21.8) of respondents agreed with this belief, while in stratum A this perception fell to 13.2% (95%CI 11.9;14.6). Confidence in vaccines distributed by the government was present in 95.4% of responses, with higher proportions in stratum A (97.0%) and in Fortaleza (98.1%); and lower in stratum C (94.3%) and in Maceió (90.3%) (Figure 2).

The decision to vaccinate their children with all vaccines available was reported by 96.9% of respondents. Three families (in Natal, Recife and São Luís) chose not to have any vaccine administered, while 265 (2.6%) decided not to administered some of the vaccines. In this latter group, when interviewees were asked about the reasons for their answer, the following justifications stood out: fear of reaction (45.3%), fear of injections (21.5%), not believing in vaccines (15, 8%), the doctor advised them not to vaccinate (12.9%), believing that vaccines are harmful (11.7%), news in the media made them drop out of the vaccination schedule (8.2%) and the belief that the disease no longer exists (5.9%).

When asked if they had difficulty taking their child to the vaccination service, 852 (8.3%) said they did. Of these, 46.6% explained that the health center was a long way away; 35.7% reported not having time to take their child to be vaccinated; 28.9% indicated that the health center opening hours were incompatible; 27.3% reported that they did not have transport; 22.8% did not have enough money; 7.4% said their employer would not give them time off; and 3.1% because they had lost their child’s vaccination card.

When asked whether their child had failed to be vaccinated despite being taken to the health center, there were 3,572 (34.7%) positive responses. The main reasons were: lack of vaccine (86.8%), closed vaccination room (23.5%), lack of material (19.4%), absence of a health professional (15.3%), health professional who recommended not administering vaccines on the same day (14.9%), there was a long line (13.7%), no more line number tickets available (12.3%), it was not the right day for that vaccine (10.6%).

In the multivariate analysis, factors associated with non-vaccination were found to be living in areas of the highest socioeconomic stratum – A – (OR-a = 1.34; 95%CI 1.20;1.50), with emphasis on people living in São Luís (OR-a = 2.78; 95%CI 2.33;3.32), use of a private vaccination service (OR-a = 2.13; 95%CI 1.87;2.42 ), mother or guardian without a paid job (OR-a = 1.11; 95%CI 1.02;1.21) and having more than one child (OR-a = 1.21; 95 %CI 1.17;1.26). There was no statistically significant association with child-related characteristics (Table 3).

**Table 3:** Sociodemographic factors associated with non-vaccination among children born in 2017-2018, in the state capital cities of the Northeast region of Brazil, Vaccination Coverage Survey 2020-2022 (n = 10,290)

Variables	Crude OR^a^ (non-vaccination) (95%CI)^b^	p-value	Adjusted OR (non-vaccination) (95%CI)^b,c^	p-value
Ecological/socioeconomic stratum		< 0.01		< 0.0001
A	1.61 (1.43;1.80)		1.34 (1.20;1.50)	
B	1.08 (0.97;1.20)		1.02 (1.01;1.19)	
C	0.91 (0.82;1.02)		1.19 (1.22;1.61)	
D	1		1	
**City**		**< 0.01**		**< 0.0001**
São Luís (MA)	2.74 (2.26;3.33)		2.78 (2.33;3.32)	
Teresina (PI)	1		1	
Fortaleza (CE)	1.40 (1.19;1.65)		1.37 (1.19;1.57)	
Natal (RN)	2.25 (1.83;2.76)		2.20 (1.82;2.65)	
João Pessoa (PB)	2.40 (1.99;2.91)		2.28 (1.92;2.70)	
Recife (PE)	1.46 (1.24;1.71)		1.38 (1.20;1.59)	
Maceió (AL)	1.69 (1.40;2.03)		1.73 (1.47;2.04)	
Aracaju (SE)	1.29 (1.07;1.55)		-	
Salvador (BA)	1.33 (1.13;1.56)		1.23 (1.07;1.41)	
**Use of private service for vaccination**		**< 0.01**		**< 0.0001**
Yes	2.18 (1.94;2.45)		2.13 (1.87;2.42)	
No	1		1	
*Bolsa Família*		**0.32**		
Yes	1		-	-
No	1.18 (1.09;1.28)		-	-
**Maternal characteristics**				
**Schooling (years of study)**		**0.41**		
0-8	1		-	-
9-12	0.88 (0.76;1.02)		-	-
13-15	0.74 (0.65;0.84)		-	-
16 or more	1.11 (0.96;1.27)		-	-
**Race/skin color**		**0.36**		
White	1.43 (1.26;1.62)		-	-
Black	1		-	-
Mixed race	1.16 (1.03;1.29)		-	-
Asian	1.27 (0.81;2.01)		-	-
Indigenous	1.30 (0.48;3.52)		-	-
**Paid job**		**< 0.01**		**< 0.0001**
Yes	1		1	
No	1.21 (1.12;1.31)		1.11 (1.02;1.21)	
**Marital status (has a partner)**		**0.47**		
Yes	1		-	-
No	0.95 (0.87;1.03)		-	-
Unable to answer/did not answer	1.36 (1.08;1.72)		-	-
**Number of children alive**		**< 0.01**		**< 0.0001**
Average	1.16 (1.12;1.20)		1.21 (1.17;1.26)	

a) Odds ratio; b) 95% confidence interval; c) Variables included in the multivariate model (variables with 0.05% significance; and collinearity below 20%): stratum, city, use of private service for vaccination, schooling (years of study) [category: ≥ 16], paid job and number of children alive.

## DISCUSSION

The findings of this study are in line with the worrying scenario of low coverage observed in Brazil.^22-24^ The targets were not achieved for any of the vaccines assessed in the Northeastern capital cities taken together. Aspects related to social vulnerability, such as a mother with more than one child and no paid work, as well as the use of private health services and belonging to the highest socioeconomic stratum were associated with lower coverage. Operational issues related to health services are an important point regarding vaccination hesitancy, in addition to misinformation about vaccines.

It is worth noting that, even before the COVID-19 pandemic, when social distancing was recommended, inadequate vaccination coverage was already being highlighted, becoming a priority on the global health agenda.^25^ In 2019, the year immediately after the birth of the children monitored in this study, Brazil had not achieved the target for any of the vaccines recommended for children under 1 year old.^11^

The reduction in vaccination coverage may reflect, among other factors, the increase in the dropout rate, which refers to people who started but did not complete the vaccination schedule. This is a worrying scenario, because receiving the initial doses may create the false impression that some immunity has been achieved; however, it is known that immunological protection will only be achieved after taking the full vaccination schedule.^26,27^

The higher proportion of non-vaccination in the highest socioeconomic stratum may be influenced by vaccination hesitancy in the population with better family income, among which parent/guardian intention to postpone vaccination or not to vaccinate their children is seen to be greater.^27,28^ Added to this is the use of private vaccination services by the population belonging to this stratum, mentioned in previous vaccination surveys in Brazil, which may compromise the monitoring of vaccination status, given the unavailability of these data on the PNI Information System.^28^ In contrast, incomplete vaccination coverage was also associated with the presence of more than one child, demonstrating the influence of the context of socioeconomic vulnerability.^29^

In addition to vaccination coverage indicators and associated factors, aspects related to vaccination hesitancy found by this study bring relevant perspectives. Although it is known that acceptance of vaccination by the general population is no longer the same, nor as obvious, as in previous decades, the findings of this survey indicate that the absolute majority of respondents (99.1%) believe that vaccines are important, countering the global trend of vaccination hesitancy. Two extreme behaviors – “receiving all doses of the vaccination schedule” and “not accepting any doses” – demonstrate heterogeneity in behavior, challenging the understanding of these complex dynamics. Currently, the Strategic Advisory Group of Experts on Immunization (SAGE) considers that, in addition to simple individual refusal, vaccination hesitancy can be present in external situations caused by structural problems, such as low availability of vaccine stock, access barriers, limited supply times and economic difficulties, among others.^16-18^

Some of the interviewees were unable to vaccinate their children, despite having gone to the vaccination service, which demonstrates that operational issues can cause access barriers. Health center opening hours are incompatible with the needs of the population, especially mothers, many of whom are breadwinners and have jobs.^30^ In the case of women who work, often on the informal labor market, having to return to the vaccination service means taking time off from work and earning less income. Even more critically, the association of non-vaccination, in Brazilian capital cities, with the absence of paid work for mothers and for those with more than one child alive leads to the understanding of the influence of contexts of social inequities on restricting access to vaccination actions, above all in the case of very young children.^29^ For the health system, this means lost opportunities for vaccination, defined as the failure to administer indicated vaccine doses in appropriate situations, when there has been any contact with the eligible person. 

To ensure that the vaccination schedule of a child under 1 year old is up-to-date, it is necessary to visit the vaccination service at least seven times. Hence the importance of broad and continuous access to vaccines, so as not to miss vaccination opportunities. It is essential to review the actions and functioning of vaccination services in order to achieve adequate vaccination coverage, taking into consideration the needs of the population.^30^

Adoption of microplanning strategies, based on heath situation analysis in the different contexts of the SUS, is considered a necessary action. Territorial recognition of risk and vulnerability situations, as well as identification of susceptible populations and health service conditions (infrastructure and available teams) must be incorporated into the planning and execution of actions.

In times of infodemics, social communication and population engagement are essential to join efforts in favor of vaccination. Information and communication actions must be incorporated into all stages of the process, seeking to translate, in a clear, attractive and precise way, the importance, safety and effectiveness of vaccines provided by the SUS.^15^

The limitations of this study are inherent to household surveys, including logistical challenges, especially in hard-to-reach areas, refusals to participate in the survey and memory biases, as these are vaccines received in the past, which can interfere with the accuracy of the replies. Due to the COVID-19 pandemic period, fieldwork was interrupted at the most critical moments of the health crisis. To overcome these setbacks, investment was made in team training, prior scheduling and communication actions regarding safety measures.

Availability of address information on the SINASC was limited in certain areas, overloading the field team when having to search for addresses in other sources. The low quality of records on vaccination cards was also a complication, both due to the lack of standardization and the incompleteness of the data. Nevertheless, this study reinforces the importance of local research, especially after the recovery of PNI strategic actions from 2023 onwards. However, it can be further deepened with the application of more robust causal models.

Vaccination coverage in children under 24 months old born in 2017 and 2018 in the capital cities of Northeast Brazil was below pre-established parameters, both as a whole and for each vaccine individually. In all capitals, there was a drastic reduction in vaccination coverage throughout the observation period. Different dimensions of social vulnerability, operational aspects of private services, access to public services and belonging to the highest socioeconomic stratum (A) were associated with lower coverage. In situations of vaccination hesitancy, operational aspects and those related to misinformation stood out.

Obstacles inherent to the ways in which vaccination services are offered require special attention. Integrated health communication and education actions are essential to minimize uncertainty about vaccines. Rethinking current immunization strategies in the SUS, in order to adapt them to regional contexts and specificities, is necessary to support more precise analyses of the challenging aspects related to low coverage and the operational aspects that permeate them.
