# Supplementary material for: Vaccination coverage, hesitancy and associated factors: a household survey of a cohort of children born in 2017 and 2018 in urban areas of state capital cities in the Brazilian Northeast
Source: Epidemiol Serv Saude. 2024 Nov 1;33(spe2):e20231298. doi: 10.1590/S2237-96222024v33e20231298.especial2.en (PMC11537179; doi:10.1590/S2237-96222024v33e20231298.especial2.en)
Supplement: Translation

# Translation: Cobertura, hesitação vacinal e fatores associados à não vacinação: inquérito domiciliar em coorte de crianças nascidas vivas em 2017 e 2018 em áreas urbanas de capitais do Nordeste brasileiro

**DOI:** 10.1590/S2237-96222024v33e20231298.especial2.pt

**Authors:** Ramon da Costa Saavedra, Martha Suely Itaparica de Carvalho Santiago, Maria da Glória Lima Cruz Teixeira, Maria Bernadete de Cerqueira Antunes, Rejane Christine de Sousa Queiroz, Luisa Helena de Oliveira Lima, Alberto Novaes Ramos, Anderson Fuentes Ferreira, Adjoane Mauricio Silva Maciel, Jaqueline Caracas Barbosa, Ana Paula França, Carla Magda Allan Santos Domingues, José Cássio de Moraes, Grupo ICV 2020, Adriana Ilha da Silva, Alberto Novaes Ramos, Ana Paula França, Andrea de Nazaré Marvão Oliveira, Antonio Fernando Boing, Carla Magda Allan Santos Domingues, Consuelo Silva de Oliveira, Ethel Leonor Noia Maciel, Ione Aquemi Guibu, Isabelle Ribeiro Barbosa Mirabal, Jaqueline Caracas Barbosa, Jaqueline Costa Lima, José Cássio de Moraes, Karin Regina Luhm, Karlla Antonieta Amorim Caetano, Luisa Helena de Oliveira Lima, Maria Bernadete de Cerqueira Antunes, Maria da Gloria Teixeira, Maria Denise de Castro Teixeira, Maria Fernanda de Sousa Oliveira Borges, Rejane Christine de Sousa Queiroz, Ricardo Queiroz Gurgel, Rita Barradas Barata, Roberta Nogueira Calandrini de Azevedo, Sandra Maria do Valle Leone de Oliveira, Sheila Araújo Teles, Silvana Granado Nogueira da Gama, Sotero Serrate Mengue, Taynãna César Simões, Valdir Nascimento, Wildo Navegantes de Araújo

**Affiliations:** 1 Universidade Federal da Bahia, Instituto de Saúde Coletiva, Salvador, BA, Brasil; 2 Universidade de Pernambuco, Faculdade de Ciências Médicas, Recife, PE, Brasil; 3 Universidade Federal do Maranhão, Departamento de Saúde Pública, São Luís, MA, Brasil; 4 Universidade Federal do Piauí, Campus Senador Helvídio Nunes de Barros, Picos, PI, Brasil; 5 Universidade Federal do Ceará, Faculdade de Medicina, Departamento de Saúde Comunitária, Fortaleza, CE, Brasil; 6 Universidade Federal do Ceará, Faculdade de Medicina, Programa de Pós-graduação em Saúde Pública, Fortaleza, CE, Brasil; 7 Faculdade de Ciências Médicas da Santa Casa de São Paulo, São Paulo, SP, Brasil; 8 Organização Pan-Americana da Saúde, Brasília, DF, Brasil; Universidade Federal do Espírito Santo, Vitória, ES, Brasil; Universidade Federal do Ceará, Departamento de Saúde Comunitária, Fortaleza, CE, Brasil; Faculdade Ciências Médicas Santa Casa de São Paulo, São Paulo, SP, Brasil; Secretaria de Estado da Saúde do Amapá, Macapá, AP, Brasil; Universidade Federal de Santa Catarina, SC, Brasil; Organização Pan-Americana da Saúde, Brasília, DF, Brasil; Instituto Evandro Chagas, Belém, PA, Brasil; Faculdade de Ciências Médicas Santa Casa de São Paulo, Departamento de Saúde Coletiva, São Paulo, SP, Brasil; Universidade Federal do Rio Grande do Norte, Natal, RN, Brasil; Universidade Federal do Ceará, Departamento de Saúde Comunitária, Fortaleza, CE, Brasil; Universidade Federal de Mato Grosso, Cuiabá, MT, Brasil; Universidade Federal do Paraná, Curitiba, PR, Brasil; Universidade Federal de Goiás, Goiânia, GO, Brasil; Universidade Federal do Piauí, Teresina, PI, Brasil; Universidade de Pernambuco, Faculdade de Ciências Médicas, Pernambuco, PE, Brasil; Instituto de Saúde Coletiva, Universidade Federal da Bahia, Salvador, BA, Brasil; Secretaria de Estado da Saúde de Alagoas, Maceió, AL, Brasil; Universidade Federal do Acre, Rio Branco, AC, Brasil; Universidade Federal do Maranhão, Departamento de Saúde Pública, São Luís, MA, Brasil; Universidade Federal de Sergipe, Aracaju, SE, Brasil; Secretaria Municipal de Saúde, Boa Vista, RR, Brasil; Fundação Oswaldo Cruz, Mato Grosso do Sul, Campo Grande, MS, Brasil; Fundação Oswaldo Cruz, Escola Nacional de Saúde Pública Sergio Arouca, Rio de Janeiro, RJ, Brasil; Universidade Federal do Rio Grande do Sul, Porto Alegre, RS, Brasil; Fundação Oswaldo Cruz, Instituto de Pesquisa René Rachou, Belo Horizonte, MG, Brasil; Secretaria de Desenvolvimento Ambiental de Rondônia, Porto Velho, RO, Brasil; Universidade de Brasília, Brasília, DF, Brasil

**Keywords:** Cobertura Vacinal, Hesitação Vacinal, Vacinação em Massa, Programas de Imunização, Inquéritos Epidemiológicos

## Abstract

**Objetivo:**

Estimar a cobertura vacinal e analisar fatores sociodemográficos associados à não vacinação em crianças nascidas vivas em 2017 e 2018 nas capitais do Nordeste brasileiro.

**Métodos:**

Realizou-se inquérito domiciliar com amostragem por conglomerados, entre 2020 e 2022, para estimar cobertura e hesitação vacinal. Fatores associados à não vacinação foram analisados usando-se regressão logística para calcular *odds ratio* (OR) e seus intervalos de confiança (IC_95_%).

**Resultados:**

A capital com menores coberturas vacinais foi Natal, com < 75,0% para a maioria dos imunizantes; Teresina apresentou coberturas ≥ 90,0% em todos os imunizantes. Dos entrevistados, 99,1% (IC_95_% 98,9;99,3) acreditavam que vacinas são importantes para a saúde; 95,4% (IC_95_% 95,0;95,8) confiavam nos imunobiológicos distribuídos pelo governo; e 79,6% (IC_95_% 78,8;80,3) não tinham medo de reações adversas. Residir em estrato socioeconômico mais alto (OR ajustado: 1,34 – IC_95_% 1,20;1,50) foi fator associado à não vacinação.

**Conclusão:**

As baixas coberturas destacam a necessidade de entender melhor as especificidades regionais e as desigualdades sociais.

## Contribuições do estudo

**Principais resultados:**

Três capitais nordestinas alcançaram metas para apenas duas ou três vacinas. A maioria dos entrevistados expressou opiniões favoráveis em relação ao Programa Nacional de Imunizações. No entanto, um terço dos entrevistados relataram ter ido em busca da vacina, mas não conseguiram vacinar suas crianças.

**Implicações para os serviços:**

É necessário repensar as estratégias de imunização no Sistema Único de Saúde, para adequá-las aos contextos regionais. Entraves operacionais relacionados à oferta das vacinas requerem atenção especial, além de ações integradas de comunicação e educação em saúde.

**Perspectivas:**

A compreensão das especificidades regionais apresentadas subsidiará o Programa Nacional de Imunizações na implementação de estratégias contextualizadas aos territórios, via microplanejamento das ações de vacinação, para resgate de coberturas vacinais.

## INTRODUÇÃO

O investimento em ações sustentáveis para ampliação das coberturas vacinais contribuiu no controle de doenças imunopreveníveis no Brasil e no mundo, reduzindo a morbimortalidade por essas infecções e ampliando a expectativa de vida.^1,2^ Na região Nordeste do país, a ampliação da Estratégia Saúde da Família e as iniciativas de transferência de renda também foram importantes para a melhoria das coberturas vacinais.^3,4^

A intensificação das ações de imunização no Nordeste brasileiro impactou positivamente as condições de saúde da população. Após a instituição do Plano Nacional de Eliminação do Sarampo (1992), o Ceará passou 13 anos sem registro de casos da doença, entre 2000 e 2013.^6,7^ A introdução da vacina oral contra o rotavírus humano (2006) reduziu as internações e os óbitos de crianças nordestinas,^7^ com maiores quedas observadas em Recife-PE (77% entre 2006 e 2007).^8^ A introdução da vacina meningocócica C (2010) impulsionou notável diminuição nos casos de meningite meningocócica por esse sorogrupo na região – aproximadamente 80%, entre 2010 e 2018.^9^

Apesar dos avanços alcançados em décadas de trabalho articulado entre as três esferas públicas de gestão do Sistema Único de Saúde (SUS), a partir de 2016, observou-se significativo declínio na cobertura vacinal dos principais imunizantes indicados ao público infantil. No Nordeste, essa redução foi ainda maior em menores de 1 ano de idade, em comparação a outras regiões brasileiras.^9-11^

Diversas hipóteses têm sido levantadas para justificar este cenário crítico, que não deve ser atribuído a um único fator. A eliminação de algumas doenças imunopreveníveis modificou a percepção de risco de considerável parcela da população. A falsa sensação de segurança contribuiu para o não reconhecimento da vacinação como intervenção necessária para proteção à saúde.^12,13^ A desinformação, associada à disseminação de notícias falsas por movimentos antivacinas, é outro fator relevante.^14^ Pesquisa recente apontou que o Brasil vive uma “epidemia de desinformação” sobre vacinas.^15^

Nesse contexto, a hesitação vacinal surge como fenômeno preocupante. Para além da simples recusa à vacinação, aspectos operacionais e estruturais dos serviços de imunização precisam ser melhor compreendidos.^16,17^ Em 2019, a Organização Mundial da Saúde (OMS) elencou dez ameaças globais à saúde e incluiu a hesitação vacinal entre as prioridades a serem trabalhadas.^18^ Compreender esses fenômenos e identificar como eles influenciam na cobertura vacinal é essencial para fornecer evidências que subsidiem o delineamento de políticas públicas efetivas e contextualizadas quanto às especificidades regionais.

Os índices de cobertura vacinal refletem a adesão da população ao programa de vacinação, a existência de pessoas sob risco de doenças imunopreveníveis e a efetividade dos serviços de saúde.^19^ Considerando-se que a realização de inquéritos para avaliação de cobertura vacinal representa uma ação relevante, este estudo objetiva estimar a cobertura vacinal e analisar os fatores sociodemográficos associados à não vacinação em crianças nascidas vivas em 2017 e 2018 nas capitais do Nordeste brasileiro.

## MÉTODOS

### Desenho do estudo

Inquérito domiciliar com amostragem por conglomerados baseado em coorte de crianças nascidas vivas em 2017 e 2018, em áreas urbanas das capitais do Nordeste do Brasil, realizado entre 2020 e 2022. Trata-se de um recorte do *Inquérito de cobertura vacinal nas capitais de 26 estados, Distrito Federal e 12 municípios do interior em crianças nascidas em 2017-2018 residentes em área urbana*, cujos detalhes dos métodos e aspectos operacionais foram apresentados em artigo.^20^

### Contexto

A região Nordeste tem extensão territorial de 1.558.000 km² e população estimada de 54.644.582 habitantes (26,9% da população do país). As capitais nordestinas possuem 11.385.286 habitantes, tendo Aracaju (602.757) a menor e Fortaleza (2.428.708) a maior população.^21^

### População e fonte de dados

A população do estudo é composta por crianças nascidas nos anos de 2017 e 2018, residentes nas nove capitais da região Nordeste, segundo o Sistema de Informação sobre Nascidos Vivos (Sinasc). A fonte de dados foi o inquérito nacional mencionado anteriormente.

### Procedimento amostral

Participaram do estudo crianças cujas trajetórias de vacinação foram analisadas desde o nascimento até os 24 meses de idade, que tiveram seus endereços, conforme o Sinasc, georreferenciados em setores censitários de residência e agrupados em conglomerados formados por quatro estratos ecológicos (A, B, C, D) definidos por características socioeconômicas, em que o A apresenta as melhores condições de vida, e o D, as piores.

Para definição dos estratos, foram utilizados os setores censitários de cada cidade, conforme o Censo Demográfico de 2010, classificados com base na renda média dos responsáveis pelo domicílio, na proporção de responsáveis alfabetizados e na proporção de responsáveis com renda maior ou igual a 20 salários mínimos.

A partir dos conglomerados, foi realizada seleção aleatória do número previsto de crianças em cada estrato. O tamanho da amostra nos estratos variou de acordo com o número de inquéritos realizados nos municípios, e foi definido segundo diretrizes estabelecidas no artigo metodológico publicado.^20^

### Coleta de dados e variáveis

As visitas domiciliares ocorreram no período de 2020 a 2022, de acordo com os endereços identificados no Sinasc. Utilizou-se questionário para a coleta de informações sobre características sociodemográficas:

- Da família: aglomeração intradomiciliar (sim/não), presença de avó/avô morando no domicílio (sim/não), acesso a benefício social (sim/não) e renda mensal (até R$ 1.000, de R$ 1.001 a R$ 3.000, de R$ 3.001 a R$ 8.000, e acima de R$ 8.000);
- Da mãe da criança: faixa etária (< 20 anos, 20-34 anos, 35 anos ou mais), raça/cor da pele (branca, preta, parda, amarela, indígena), escolaridade (0 a 8 anos de estudo, 9 a 12 anos, 13 a 15 anos, 16 anos ou mais), existência de trabalho (sim/não), viver com o companheiro (sim/não), número de filhos vivos;
- Da criança: sexo (masculino/feminino), raça/cor da pele, frequenta creche (sim/não), tipo de parto (normal/cesariana), ordem de nascimento.

Sobre a imunização, foram coletadas informações referentes ao tipo de vacina e ao número de doses recebidas. O esquema vacinal completo considerado neste estudo, até 24 meses de vida, foi o mesmo preconizado pelo Programa Nacional de Imunizações (PNI), que inclui a administração das seguintes vacinas: bacilo de Calmette-Guérin (BCG) e hepatite B, ao nascer; pentavalente (contra difteria, tétano, pertússis, hepatite B e *Haemophilus influenzae B*) e vacina poliomielite inativada (VIP), aos 2, 4 e 6 meses; pneumocócica 10-valente e vacina rotavírus humano, aos 2 e 4 meses; vacina contra febre amarela, aos 9 meses; vacina conjugada meningocócica C, aos 3, 5 e 12 meses; vacina tríplice viral (contra sarampo, caxumba e rubéola), aos 12 e 15 meses; e vacina adsorvida difteria, tétano, pertússis (DTP), hepatite A, poliomielite oral (VOP) e varicela, aos 15 meses de vida.

A hesitação vacinal foi analisada na perspectiva dos responsáveis pelas crianças, abordando-se questões sobre crenças (confiança nas vacinas), medo de reações, importância da vacinação e decisão de vacinar (ou não). Essas respostas foram interpretadas como desfavoráveis, indiferentes ou favoráveis às ações propostas pelo PNI. Também foram levantados quesitos de ordem pessoal (dificuldades para levar a criança a se vacinar) e operacional (barreiras de acesso aos serviços) que poderiam dificultar a vacinação.

Por fim, verificou-se a situação vacinal por meio do registro fotográfico da caderneta de vacinação da criança.

### Métodos estatísticos

Os indicadores de coberturas vacinais foram calculados considerando-se o número total de últimas doses do esquema para cada imunizante recebido (numerador) e a população amostral (denominador), multiplicando-se por 100. Como referência, foram utilizadas as metas do Calendário Nacional de Vacinação da Criança, a saber: 90% para vacinas BCG e vacina rotavírus humano; 95% para as demais vacinas. Neste estudo, as análises não consideraram a vacina contra febre amarela, porque em 2017 e 2018 não havia recomendação desse imunobiológico para a maioria dos estados do Nordeste. 

Estimativas ponderadas de cobertura vacinal e intervalos de confiança (IC_95_%) foram calculados para cada vacina, utilizando-se significância estatística de p < 0,05.^21^

Foi realizada análise exploratória para a identificação de fatores de risco associados à não vacinação, por meio de regressão logística, com cálculo da razão de chance – *odds ratio* (ORc) – e intervalos de confiança de 95% (IC_95_%). Variáveis que apresentaram associação com p < 0,20 na regressão univariada foram incluídas no modelo, com o cálculo da OR ajustada (OR-a), utilizando-se o método *stepwise*. A colinearidade do modelo foi verificada por análise do fator de inflação da variância, com exclusão do modelo das variáveis com sua presença. Para a variável dependente – *status* de vacinação aos 24 meses de idade –, procedeu-se à dicotomização em *completamente vacinada* (referência), considerando-se crianças com todas as doses do esquema vacinal, ou *sem vacinação completa*, para aquelas com ausência de uma ou mais doses válidas do esquema vacinal.

As análises foram conduzidas por meio do *software* R® versão 4.2.2, utilizando-se o pacote tidyverse; Stata® versão 13 e Microsoft Office Excel®.

### Considerações éticas

O estudo foi aprovado pelos Comitês de Ética em Pesquisa com Seres Humanos do Instituto de Saúde Coletiva da Universidade Federal da Bahia, sob parecer n^o^ 3.366.818, em 4 de junho de 2019, com Certificado de Apresentação de Apreciação Ética (CAAE) 4306919.5.0000.5030; e da Irmandade da Santa Casa de São Paulo, sob parecer n^o^ 4.380.019, em 4 de novembro de 2020, com CAAE 39412020.0.0000.5479.

## RESULTADOS

Foram realizadas 10.290 entrevistas, distribuídas proporcionalmente entre estratos socioeconômicos e cidades. Desse total, 2.249 (21,9%) pertenciam ao estrato A; 2.659 (25,8%), ao B; 2.677 (26,0%), ao C; e 2.705 (26,3%), ao D. O maior número de entrevistas ocorreu em Salvador (1.818; 17,7%), Recife (1.689; 16,4%) e Fortaleza (1.612; 15,7%); e o menor, em Natal (685; 6,7%). A caderneta de vacinação foi apresentada por 99,2% das famílias. O uso dos serviços privados de vacinação pelo menos uma vez foi identificado em 15,0% das entrevistas (Tabela 1).

**Tabela 1:** Proporção (%) das características sociodemográficas das famílias de crianças nascidas em 2017-2018, segundo as capitais dos estados da região Nordeste, Inquérito de Cobertura Vacinal 2020-2022 (n = 10.290)

**Características**	**Aracaju**	**Fortaleza**	**João Pessoa**	**Maceió**	**Natal**	**Recife**	**Salvador**	**São Luís**	**Teresina**	**Total**
**Da família**										
**Aglomeração no domicílio**	5,0	9,8	4,3	8,6	8,0	9,9	12,2	8,4	13,6	9,3
**Beneficiário do Bolsa Família**	23,1	51,7	28,9	40,4	31,2	32,6	36,6	36,3	32,0	36,0
**Presença de avó no domicílio**	47,8	41,1	31,1	37,1	55,0	42,3	36,8	63,7	73,6	30,8
**Renda familiar mensal**										
Até R$ 1.000,00	18,0	38,8	21,0	46,8	36,9	37,8	45,2	29,6	14,7	34,1
De R$ 1.001,00 a R$ 3.000,00	31,1	43,3	46,9	23,5	32,6	24,9	30,9	37,9	43,8	34,4
De R$ 3.001,00 a R$ 8.000,00	24,8	9,4	20,1	12,8	16,1	13,0	8,0	13,3	18,2	13,9
R$ 8.001,00 ou mais	13,9	3,8	11,1	5,3	6,3	9,4	9,4	7,8	13,0	8,7
Não sabe/não respondeu	12,2	4,7	0,9	11,6	8,2	14,9	6,4	11,2	10,2	8,9
**Da mãe**										
**Escolaridade (anos de estudo)**										
0 a 8	9,4	10,1	8,1	19,5	13,7	12,1	13,3	5,4	10,3	11,5
9 a 12	12,7	25,5	12,9	18,6	13,9	16,5	20	11,4	13,7	17,2
13 a 15	39,6	47,5	41,4	40,2	38,7	45,9	41,4	50,4	41,2	43,4
16 ou mais	33,7	14,3	36,4	17,4	31,8	23,7	22,7	27,2	33	25,1
Não sabe/não respondeu	4,7	2,7	1,2	4,3	1,9	1,8	2,6	5,7	1,8	2,8
**Idade ao nascimento da criança (em anos)**										
< 20	3,1	2,5	3,4	5,7	2,2	3,3	2,4	3	3,4	3,1
20 a 34	58,4	64,5	56,3	70,4	59,6	62,5	56,5	67	61,3	61,6
35 ou mais	38,2	30,9	40,2	23,5	38,2	33,9	40,9	29,6	34,8	34,7
Não sabe/não respondeu	0,2	2,1	0,1	0,4	0	0,2	0,2	0,4	0,4	50
**Raça/cor da pele**										
Branca	23,6	20,2	34,2	36,9	40,9	30,8	13,4	23,7	21,6	25,5
Preta	12,8	5,5	4,3	8,5	12,3	12,8	40	16,5	16	15,9
Parda	57,6	71,5	59,2	52,4	43,5	54,2	42,3	54,2	60,5	55,2
Amarela	1,2	0,4	0,9	0,2	1,5	0,4	1,7	0,5	0	0,8
Indígena	0,2	0,1	0,2	0,2	0,1	0,2	0,2	0,1	0	20
Não sabe/não respondeu	4,7	2,3	1,2	1,7	1,8	1,6	2,5	5	1,9	2,4
**Possui trabalho remunerado**	56,1	52	46,8	41	47,7	41,8	46,9	43,8	50,2	47,2
**Vive com o companheiro**	70,6	70,5	80,6	66,2	70,5	68	65,1	67,9	70,5	69,4
**Número de filhos (média)**	1,89	2,01	2,09	2,02	1,98	2,07	2,06	1,96	1,98	2,02
**Da criança**										
**Sexo**										
Masculino	51,6	53,1	51,2	50,2	53,9	49,6	51,6	52,8	48,3	51,3
Feminino	48,4	46,9	48,8	49,8	46,1	50,4	48,4	47,2	51,7	48,7
**Ordem de nascimento**										
Primeiro	52,8	48,3	50,6	48,2	49,6	47	49,4	50,5	49,6	49,3
Segundo	29,6	31,9	29,1	33,6	29,6	31,1	28,7	31,6	32,3	30,8
Terceiro	10,9	13,3	12,8	12,5	13,1	13,1	12,2	11,4	11,8	12,4
Quarto ou mais	6,3	6,5	7,5	5,5	7,6	8,8	9,7	6,6	6,3	7,5
**Raça/cor da pele**										
Branca	33	30,4	40,5	39,8	55,6	41,3	19,3	33,3	30,9	34,1
Preta	7,8	2,9	1,5	4,1	4,4	6	30,1	8,8	10,8	9,9
Parda	57,8	66,4	57,3	55,9	39,3	52,2	48,6	57,6	58,2	55,2
Amarela	1	0,2	0,3	0,2	0,6	0,4	1,8	0,2	0	60
Indígena	0,2	0,1	0,1	0	0	0	0	0,1	0	0
Não sabe/não respondeu	0,2	0	0,2	0	0,1	0,1	0,3	0	0,1	10
Frequenta creche/escola	45	44,4	29,2	27,6	46,3	22,8	31,3	27,5	27,5	33
**Tipo de parto da criança**										
Normal (vaginal)	43,8	40,6	34,5	39,5	35,6	49,4	52,9	43,2	33,6	43,1
Cesariana	56	59,2	65,4	59,5	64,4	50,5	47	56,6	66,4	56,7
Não sabe	0,2	0,2	0,1	1	0	0,1	0,1	0,2	0	0,2
**Possui caderneta de vacinação**	99,6	99,3	99,6	97,4	99,4	98,8	99,3	99,2	100	99,2
**Usou serviço privado**	23,7	11,5	15,9	7,4	19,1	15,7	14,2	16,7	14,9	15

Constatou-se que 9,3% dos domicílios tinham aglomeração intradomiciliar (mais de três pessoas por dormitório), e 36,0% das famílias tinham acesso a benefício social. Renda mensal familiar de até R$ 1.000,00 foi verificada em 34,1% dos entrevistados, com maior proporção no estrato D (53,4%) e menor no A (12,9%). Maceió teve maior parcela de famílias nessa faixa de renda (46,8%). Apenas 5,7% referiram renda mensal maior que R$ 8.000,00, concentradas no estrato A (29,7%) e em Aracaju (13,9%) (Tabela 1).

Mães de raça/cor da pele parda ou preta (71,1%) predominaram, variando de 55,8% (Natal) a 82,3% (Salvador). Mães brancas foram mais frequentemente referidas no estrato A (40,0%), e as de raça/cor da pele preta, no estrato D (20,1%). A maioria das mães (43,4%) tinham entre 13 e 15 anos de estudos concluídos, principalmente nos estratos C (52,0%) e D (47,7%) (Tabela 1).

A maioria das crianças era do sexo masculino (52,3%), principalmente nos estratos B e C. No estrato A (50,5%) e nas cidades de Teresina (51,7%) e Recife (50,4%), houve maioria feminina. Crianças pardas (55,2%) predominaram no geral, com Fortaleza apresentando 66,4%. A maior proporção de crianças brancas se verificou em Natal (55,6%), acima do percentual geral (34,1%) (Tabela 1).

Na análise de cobertura vacinal, quatro capitais conseguiram alcançar a meta para pelo menos um imunobiológico: Recife [BCG, 93,2% (IC95% 91,7;94,7)]; Salvador [BCG, 95,3% (IC_95_% 94,4;96,38); pneumocócica 10, 95,4% (IC_95_% 94,4;96,3)], São Luís [BCG, 95,1% (IC_95_% 93,6;96,6); pneumocócica 10, 95,2% (IC_95_% 93,7;96,6)] e Teresina [BCG, 94,9% (IC95% 93,4;96,3); pneumocócica 10, 97,3% (IC_95_% 96,3;98,4); rotavírus humano, 90,3% (IC95% 88,4;92,3); meningocócica C, 96,0% (IC_95_% 94,7;97,2); tríplice viral, 97,1% (IC_95_% 96,0;98,2); hepatite A, 95,6% (IC95% 94,2;97,0)]. As demais capitais não alcançaram as metas para nenhuma das vacinas. Natal apresentou menores coberturas vacinais, com média geral de 77,3%; por outro lado, Teresina teve os melhores indicadores, tanto na média conjunta (93,9%) quanto na análise por vacina, sendo a única cidade onde todos os imunizantes apresentaram coberturas vacinais maiores ou iguais a 90% (Tabela 2).

**Tabela 2:** Coberturas vacinais (%) e intervalos de confiança de 95% de vacinas em crianças nascidas em 2017-2018 nas capitais dos estados da região Nordeste do Brasil, Inquérito de Cobertura Vacinal 2020-2022 (n = 10.290)

	**Brasil** ^a^	**Aracaju**	**Fortaleza**	**João Pessoa**	**Maceió**	**Natal**	**Recife**	**S** **ão Luís**	**Salvador**	**Teresina**
**Dose única do bacilo Calmette-Guérin**	89.6 (89.0;90.2)	85.3 (82.8;87.8)	87.5 (85.6;89.4)	89.5 (87.1;91.9)	86.5 (84.0;89.1)	70.9 (67.7;74.2)	93.2 (91.7;94.7)	95.1 (93.6;96.6)	95.3 (94.4;96.3)	94.9 (93.4;96.3)
**Dose única da hepatite B**	88.7 (88.1;89.3)	85.5 (83.1;87.9)	86.1 (84.3;87.9)	88.7 (86.4;91.0)	83.2 (80.7;85.7)	70.8 (67.6;74.2)	92.7 (91.3;94.0)	94.2 (92.6;95.7)	93.7 (92.6;94.8)	94.3 (92.8;95.8)
**Segunda dose da pneumocócica**	90.3 (89.7;90.9)	87.4 (85.2;89.6)	87.9 (86.0;89.8)	88.8 (86.5;91.1)	86.2 (83.7;88.7)	86.6 (83.5;89.7)	91.8 (90.5;93.1)	95.2 (93.7;96.6)	95.4 (94.4;96.3)	97.3 (96.3;98.4)
**Segunda dose da rotavírus humano**	82.0 (81.3;82.7)	84.3 (81.9;86.7)	77.7 (75.7;79.9)	76.1 (73.5;78.8)	76.4 (73.8;79.0)	68.3 (64.9;71.7)	86.0 (84.3;87.7)	82.4 (79.9;85.0)	85.1 (83.5;86.7)	90.3 (88.4;92.3)
**Segunda dose da meningocócica C**	89.3 (88.7;89.9)	87.8 (85.6;90.0)	85.6 (83.7;87.4)	87.0(84.5;89.5)	85.8 (83.4;88.2)	84.3 (81.1;87.4)	89.9 (88.5;91.3)	94.0 (92.4;95.6)	94.9 (93.8;95.9)	96.0 (94.7;97.2)
**Terceira dose da pentavalente**	87.9 (87.3;88.5)	86.9 (84.7;89.1)	83.5 (81.6;85.4)	83.7 (81.1;86.3)	83.9 (81.5;86.3)	76.2 (73.0;79.4)	90.1 (88.7;91.5)	91.8 (89.9;93.6)	94.0 (92.9;95.1)	92.5 (90.8;94.3)
**Terceira dose da poliomielite**	87.8 (87.2;88.4)	87.1 (84.8;89.4)	83.7 (81.8;85.6)	86.1 (83.6;88.6)	85.3 (82.8;87.8)	73.7 (70.6;76.8)	85.1 (83.3;86.7)	94.0 (92.4;95.6)	94.0 (92.9;95.1)	92.9 (91.2;94.6)
**Primeira dose da tríplice viral**	90.8 (90.2;91.4)	88.4 (86.1;90.7)	88.1 (86.3;90.0)	89.5 (87.3;91.8)	84.1 (81.6;86.6)	84.1 (80.9;87.3)	92.3 (91.0;93.6)	92.5 (90.7;94.3)	94.6 (93.6;95.6)	97.1 (96.0;98.2)
**Dose única da hepatite A**	88.1 (87.5;88.7)	86.3 (84.0;88.7)	86.0(84.1-87.9)	83.8 (81.4-86.2)	82.9 (80.4-85.4)	81.4 (78.2-84.6)	88.4 (86.9-89.9)	86.5 (84.2-88.8)	92.4 (91.2-93.6)	95.6 (94.2-97.0)
**Reforço da difteria, tétano e pertússis**	83.9 (83.2;84.6)	83.6 (81.2;85.9)	78.8 (76.6;81.0)	75.1 (72.5;77.7)	76.6 (74.0;79.2)	74.3 (71.2;77.5)	84.3 (82.6;86.0)	84.7 (82.2;87.1)	91.7 (90.4;93.0)	89.9 (87.9;91.8)
**Dose única da varicela**	86.9 (86.2;87.6	86.3 (84.1;88.4)	85,0 (83.1;86.9)	79.9 (77.4;82.4)	82.9 (80.4;85.4)	79.4 (76.2;82.6)	88.5 (87.0;90.0)	82.7 (80.1;85.2)	91.4 (90.1;92.7)	92.5 (90.8;94.3)

a) Média das capitais do Brasil e do Distrito Federal. Meta alcançada (90% para BCG e rotavírus; 95% para as demais) Meta não alcançada (90% a 94,9%, excluindo BCG e rotavírus) Meta não alcançada (85% a 89,9%) Meta não alcançada (80% a 84,9%) Meta não alcançada (75% a 79,9%) Meta não alcançada (70% a 74,9%) Meta não alcançada (abaixo de 70%)

Na evolução das coberturas vacinais, consoante sequência prevista no calendário vacinal, percebeu-se um comportamento heterogêneo entre as capitais, sendo que três delas (Teresina, Salvador e Aracaju) apresentaram desempenho superior à média nacional; outras três (Fortaleza, João Pessoa e Natal) se comportaram abaixo; e as demais (Recife, São Luís e Aracaju) situavam-se na mesma faixa das coberturas vacinais nacionais. Natal foi notoriamente a capital com menor desempenho, iniciando com 70,9% para BCG e encerrando com 36,6% para varicela (Figura 1).

**Figura 1:** Evolução das coberturas vacinais em crianças nascidas em 2017-2018, segundo vacinas e capitais dos estados da região Nordeste do Brasil, Inquérito de Cobertura Vacinal 2020-2022 (n = 10.290)

Na percepção dos pais/responsáveis para questões alusivas à hesitação vacinal, 99,1% (IC_95_% 98,9;99,3) acreditavam que as vacinas são importantes para a saúde das crianças, com pouca variação entre os estratos. Sobre a necessidade de tomar vacinas para doenças que talvez não existam mais, 80,7% (IC_95_% 79,9;81,4) consideravam necessário manter a vacinação. Uma proporção de 98,6% (IC_95_% 98,3;98,7) dos pais/responsáveis concordou com a afirmação de que “vacinar a criança é importante para a saúde das crianças do bairro”, sem variação significativa entre estratos e capitais.

Sobre a possibilidade de ocorrerem reações adversas após a administração de vacinas, 20,4% (IC_95_% 19,6;21,2) dos entrevistados disseram acreditar que as reações ocorrem por causa dos imunizantes. No estrato D, 20,2% (IC_95_% 18,8;21,8) dos respondentes concordaram com essa crença, enquanto no estrato A essa percepção se reduziu para 13,2% (IC_95_% 11,9;14,6). A confiança nas vacinas distribuídas pelo governo esteve presente em 95,4% das respostas, com maiores proporções no estrato A (97,0%) e em Fortaleza (98,1%); e menores no estrato C (94,3%) e em Maceió (90,3%) (Figura 2).

**Figura 2:** Percepção sobre as vacinas, segundo pais e responsáveis por crianças nascidas vivas em 2017 e 2018, nas capitais dos estados da região Nordeste, Inquérito de Cobertura Vacinal 2020-2022 (n = 10.290)

A decisão de aplicar todas as vacinas ofertadas foi relatada por 96,9% dos respondentes. Três famílias (em Natal, Recife e São Luís) optaram por não aplicar nenhuma vacina, ao passo que 265 (2,6%) decidiram não aplicar algumas vacinas. Nesse último grupo, ao serem os entrevistados perguntados sobre os motivos para a resposta, destacaram-se as seguintes justificativas: medo de reação (45,3%), medo de injeções (21,5%), não acreditam em vacinas (15,8%), o(a) médico(a) orientou a não tomar (12,9%), acham que vacinas fazem mal (11,7%), notícias veiculadas na mídia fizeram desistir (8,2%) e a doença não existe mais (5,9%).

Quando questionados se tiveram dificuldade para levar a criança ao serviço de vacinação, 852 (8,3%) disseram que sim. Desses, 46,6% justificaram que a unidade de saúde ficava longe; 35,7% relataram não ter tempo para levar a criança; 28,9% indicaram que o horário de funcionamento da unidade era incompatível; 27,3% relataram que não tinham transporte; 22,8%, que não tinham dinheiro; 7,4%, que o empregador não liberava; e 3,1%, porque perderam o cartão de vacinação.

Para o questionamento sobre se a criança já deixou de ser vacinada apesar de ter sido levada à unidade de saúde, houve 3.572 (34,7%) respostas positivas. Os principais motivos foram: falta de vacina (86,8%), sala de vacina fechada (23,5%), faltava material (19,4%), ausência de profissional de saúde (15,3%), profissional recomendou não aplicar vacinas no mesmo dia (14,9%), muita fila (13,7%), acabou a senha (12,3%), não era dia daquela vacina (10,6%).

Na análise multivariada, foram encontrados, como fatores associados à não vacinação, ser residente em áreas do estrato socioeconômico mais alto – A – (OR-a = 1,34; IC_95_% 1,20;1,50), com destaque para residentes em São Luís (OR-a = 2,78; IC_95_% 2,33;3,32), uso de serviço privado de vacinação (OR-a = 2,13; IC_95_% 1,87;2,42), mãe ou responsável sem trabalho remunerado (OR-a = 1,11; IC_95_% 1,02;1,21) e ter mais de um(a) filho(a) (OR-a = 1,21; IC_95_% 1,17;1,26). Não foi verificada associação estatisticamente significante com características relacionadas à criança (Tabela 3).

**Tabela 3:** Fatores sociodemográficos associados à não vacinação em crianças nascidas em 2017-2018, nas capitais dos estados da região Nordeste do Brasil, Inquérito de Cobertura Vacinal 2020-2022 (n = 10.290)

Variáveis	OR^a^ bruto (não vacinação) (IC_95_ **%**)^b^	p-valor	OR ajustada (não vacinação) (IC_95_ **%**)^b,c^	p-valor
Estrato ecológico/socioeconômico		< 0,01		< 0,0001
A	1,61 (1,43;1,80)		1,34 (1,20;1,50)	
B	1,08 (0,97;1,20)		1,02 (1,01;1,19)	
C	0,91 (0,82;1,02)		1,19 (1,22;1,61)	
D	1		1	
**Cidade**		**< 0,01**		**< 0,0001**
São Luís (MA)	2,74 (2,26;3,33)		2,78 (2,33;3,32)	
Teresina (PI)	1		1	
Fortaleza (CE)	1,40 (1,19;1,65)		1,37 (1,19;1,57)	
Natal (RN)	2,25 (1,83;2,76)		2,20 (1,82;2,65)	
João Pessoa (PB)	2,40 (1,99;2,91)		2,28 (1,92;2,70)	
Recife (PE)	1,46 (1,24;1,71)		1,38 (1,20;1,59)	
Maceió (AL)	1,69 (1,40;2,03)		1,73 (1,47;2,04)	
Aracaju (SE)	1,29 (1,07;1,55)		-	
Salvador (BA)	1,33 (1,13;1,56)		1,23 (1,07;1,41)	
**Uso de serviço privado para a vacinação**		**< 0,01**		**< 0,0001**
Sim	2,18 (1,94;2,45)		2,13 (1,87;2,42)	
Não	1		1	
Bolsa Família		0,32		
Sim	1		-	-
Não	1,18 (1,09;1,28)		-	-
**Características da mãe**				
**Escolaridade (anos de estudo)**		**0,41**		
0 a 8	1		-	-
9 a 12	0,88 (0,76;1,02)		-	-
13 a 15	0,74 (0,65;0,84)		-	-
16 ou mais	1,11 (0,96;1,27)		-	-
**Raça/cor da pele**		**0,36**		
Branca	1,43 (1,26;1,62)		-	-
Preta	1		-	-
Parda	1,16 (1,03;1,29)		-	-
Amarela	1,27 (0,81;2,01)		-	-
Indígena	1,30 (0,48;3,52)		-	-
**Trabalho remunerado**		**< 0,01**		**< 0,0001**
Sim	1		1	
Não	1,21 (1,12;1,31)		1,11 (1,02;1,21)	
**Situação conjugal (com companheiro)**		**0,47**		
Sim	1		-	-
Não	0,95 (0,87;1,03)		-	-
Não sabe/não respondeu	1,36 (1,08;1,72)		-	-
**Número de filhos vivos**		**< 0,01**		**< 0,0001**
Média	1,16 (1,12;1,20)		1,21 (1,17;1,26)	

a) *Odds ratio*; b) Intervalo de confiança de 95%; c) Variáveis inclusas no modelo multivariado (variáveis com significância de 0,05%; e colinearidade abaixo de 20%): estrato, cidade, uso de serviço privado para a vacinação, escolaridade (anos de estudo) [categoria: ≥ 16], trabalho remunerado e número de filhos vivos.

## DISCUSSÃO

Os achados deste estudo vão ao encontro do preocupante cenário de baixas coberturas observadas no país.^22-24^ Não houve alcance das metas para nenhuma das vacinas avaliadas no conjunto das capitais nordestinas. Aspectos relativos à vulnerabilidade social, tais como mãe com mais de um filho e sem trabalho remunerado, bem como a utilização de serviços privados de saúde e pertencer ao estrato socioeconômico mais alto foram associados à menor cobertura. Questões operacionais relacionadas aos serviços de saúde representam importante ponto concernente à hesitação vacinal, além da desinformação sobre vacinas.

Convém observar que, mesmo antes da pandemia de covid-19, quando houve recomendação do distanciamento social, coberturas vacinais inadequadas já vinham sendo evidenciadas, tornando-se pauta prioritária na agenda global da saúde.^25^ Em 2019, ano imediatamente posterior ao período de nascimento das crianças observadas neste estudo, o país não havia atingido a meta para nenhuma das vacinas indicadas aos menores de 1 ano.^11^

A redução das coberturas vacinais pode ser reflexo, entre outros fatores, do aumento da taxa de abandono, que se refere a pessoas que iniciaram, mas não concluíram o esquema de vacinação. Esse é um cenário preocupante, porque o recebimento das doses iniciais pode gerar a falsa impressão de que alguma imunidade foi alcançada; entretanto, sabe-se que a proteção imunológica somente estará conferida após o esquema vacinal completo.^26,27^

A maior proporção de não vacinação no estrato socioeconômico mais alto pode estar sendo influenciada pela hesitação vacinal na população com melhor renda familiar, em que se percebe maior intenção dos pais/responsáveis no adiamento ou não vacinação de seus filhos.^27,28^ Soma-se a isso o uso do serviço privado de vacinação pela população pertencente a esse estrato, citado em inquéritos vacinais anteriores no Brasil, o que pode comprometer o monitoramento da situação vacinal, haja vista a indisponibilidade desses dados no Sistema de Informação do PNI.^28^ Em contraposição, a cobertura vacinal incompleta também foi associada à presença de mais de um filho, demonstrando a influência do contexto de vulnerabilidade socioeconômica.^29^

Além dos indicadores de cobertura vacinal e fatores associados, aspectos relacionados à hesitação vacinal obtidos no estudo trazem perspectivas relevantes. Embora se saiba que a aceitação da vacinação pela população em geral já não é a mesma, e nem tão óbvia, quanto em décadas anteriores, os achados deste inquérito indicam que a maioria absoluta dos entrevistados (99,1%) acredita que vacinas são importantes, contrariando a tendência mundial de hesitação vacinal. Dois comportamentos extremos – “receber todas as doses do calendário vacinal” e “não aceitar nenhuma dose” – demonstram heterogeneidade de comportamentos, desafiando a compreensão dessa complexa dinâmica. Atualmente, o Grupo Consultivo Estratégico de Especialistas (SAGE) considera que, além da simples recusa pessoal, a hesitação vacinal pode estar presente em situações externas causadas por problemas estruturais, como baixa disponibilidade de estoque de vacinas, barreiras de acesso, horários limitados de oferta e dificuldades econômicas, entre outros.^16-18^

Parte dos entrevistados não conseguiu vacinar a criança, apesar de ter ido ao serviço, o que demonstra que questões operacionais podem ocasionar barreiras de acesso. Há horários de funcionamento das unidades incompatíveis com as necessidades da população, sobretudo de mães, muitas delas arrimo de família e vinculadas ao mercado de trabalho.^30^ Para a usuária trabalhadora, muitas vezes na informalidade, ter que retornar ao serviço de vacinação representa mais um turno afastado da sua fonte de renda. Ainda de forma mais crítica, a associação da não vacinação, nas capitais brasileiras, à ausência do trabalho remunerado para as mães e para aquelas com mais de um filho remete à compreensão da influência dos contextos de iniquidades sociais na restrição do acesso às ações de vacinação, principalmente para a população infantil.^29^ Para o sistema de saúde, isso significa perda de oportunidade de vacinação, definida como a não administração de doses de vacinas indicadas em situações adequadas, quando houve qualquer contato com a pessoa elegível. 

Para garantir esquema de vacinação atualizado em uma criança menor de 1 ano de idade, é necessário ir ao serviço de vacinação pelo menos sete vezes. Daí a importância do acesso amplo e contínuo às vacinas, a fim de não se perderem oportunidades de vacinação. É imprescindível rever as ações e o funcionamento dos serviços de vacinação para o alcance de coberturas vacinais adequadas, considerando-se as necessidades da população.^30^

A adoção de estratégias de microplanejamento, a partir do diagnóstico situacional nos diferentes contextos do SUS, é considerada uma ação necessária. O reconhecimento territorial de situações de risco e vulnerabilidade, bem como a identificação da população susceptível e das condições do serviço (infraestrutura e equipes disponíveis), devem ser incorporados ao planejamento e à execução das ações.

Em tempos de infodemia, a comunicação social e o engajamento da população são fundamentais para somar esforços em prol da vacinação. Ações de informação e comunicação devem ser incorporadas em todas as etapas do processo, buscando traduzir, de forma clara, atraente e precisa, a importância, segurança e efetividade das vacinas disponibilizadas pelo SUS.^15^

As limitações deste estudo são inerentes aos inquéritos domiciliares, incluindo desafios logísticos, especialmente em áreas de difícil acesso, recusas em participar da pesquisa e vieses de memória, por se tratar de vacinas recebidas no passado, o que pode interferir na precisão das respostas. Por ter compreendido o período pandêmico da covid-19, a execução do trabalho de campo sofreu interrupções nos momentos mais críticos da crise sanitária. Para superação desses reveses, investiu-se em treinamento da equipe, agendamento prévio e ações de comunicação acerca das medidas de segurança.

A disponibilidade de informações referentes ao endereço no Sinasc estava limitada em determinadas áreas, sobrecarregando a equipe de campo na busca por endereços em outras fontes. A baixa qualidade dos registros nas cadernetas de vacinação também foi um dificultador, tanto pela inexistência de padronização quanto pela incompletude dos dados. Ainda assim, este estudo reforça a importância de pesquisas locais, principalmente após o resgate de ações estratégicas do PNI a partir de 2023. Todavia, ele pode ser posteriormente aprofundado com a aplicação de modelos causais mais robustos.

A cobertura vacinal em menores de 24 meses nascidos nos anos de 2017 e 2018, nas capitais do Nordeste brasileiro, esteve abaixo dos parâmetros preestabelecidos, tanto no conjunto quanto para cada vacina. Em todas as capitais, houve drástica redução da cobertura vacinal, ao longo do período de observação. Diferentes dimensões de vulnerabilidade social, aspectos operacionais dos serviços privados, acesso aos serviços públicos e pertencer ao estrato socioeconômico mais alto (A) estiveram associados à menor cobertura. Nas situações de hesitação vacinal, destacaram-se aspectos operacionais e relacionados à desinformação.

Entraves inerentes às formas de oferta do serviço de vacinação requerem atenção especial. Ações integradas de comunicação e educação em saúde são imprescindíveis para minimizar incertezas acerca das vacinas. Repensar as atuais estratégias de imunização no SUS, a fim de adequá-las aos contextos e às especificidades regionais, se faz necessário para subsidiar análises mais precisas sobre os desafiadores aspectos atinentes às baixas coberturas e aos aspectos operacionais que as permeiam.
